# Supplementary material for: Neddylation regulates the development and function of glutamatergic neurons
Source: Commun Biol. 2025 Sep 9;8:1338. doi: 10.1038/s42003-025-08680-x (PMC12420789; doi:10.1038/s42003-025-08680-x)
Supplement: Supplementary file 1 — Supplementary Information [file 42003_2025_8680_MOESM1_ESM.docx]

Neddylation regulates the development and function of glutamatergic neurons

Josefa Torres^1^, Zehra Vural^1^, Maksims Fiosins^2^, Valentin Schwarze^1^, Inés Hojas-García-Plaza^1^, Fritz Benseler^1^, Stefan Bonn^2^, Silvio O. Rizzoli^3^, Benjamin H. Cooper^1^, JeongSeop Rhee^1^, Nils Brose^1^, Marilyn Tirard^1^

^1^Department of Molecular Neurobiology, Max Planck Institute for Multidisciplinary Sciences, Göttingen, Germany

^2^Institute of Medical Systems Biology, Center for Biomedical AI (bAIome), Center for Molecular Neurobiology (ZMNH), University Medical Center Hamburg-Eppendorf, Hamburg, Germany

^3^Department for Neuro- and Sensory Physiology, University Medical Center Göttingen, Göttingen, Germany


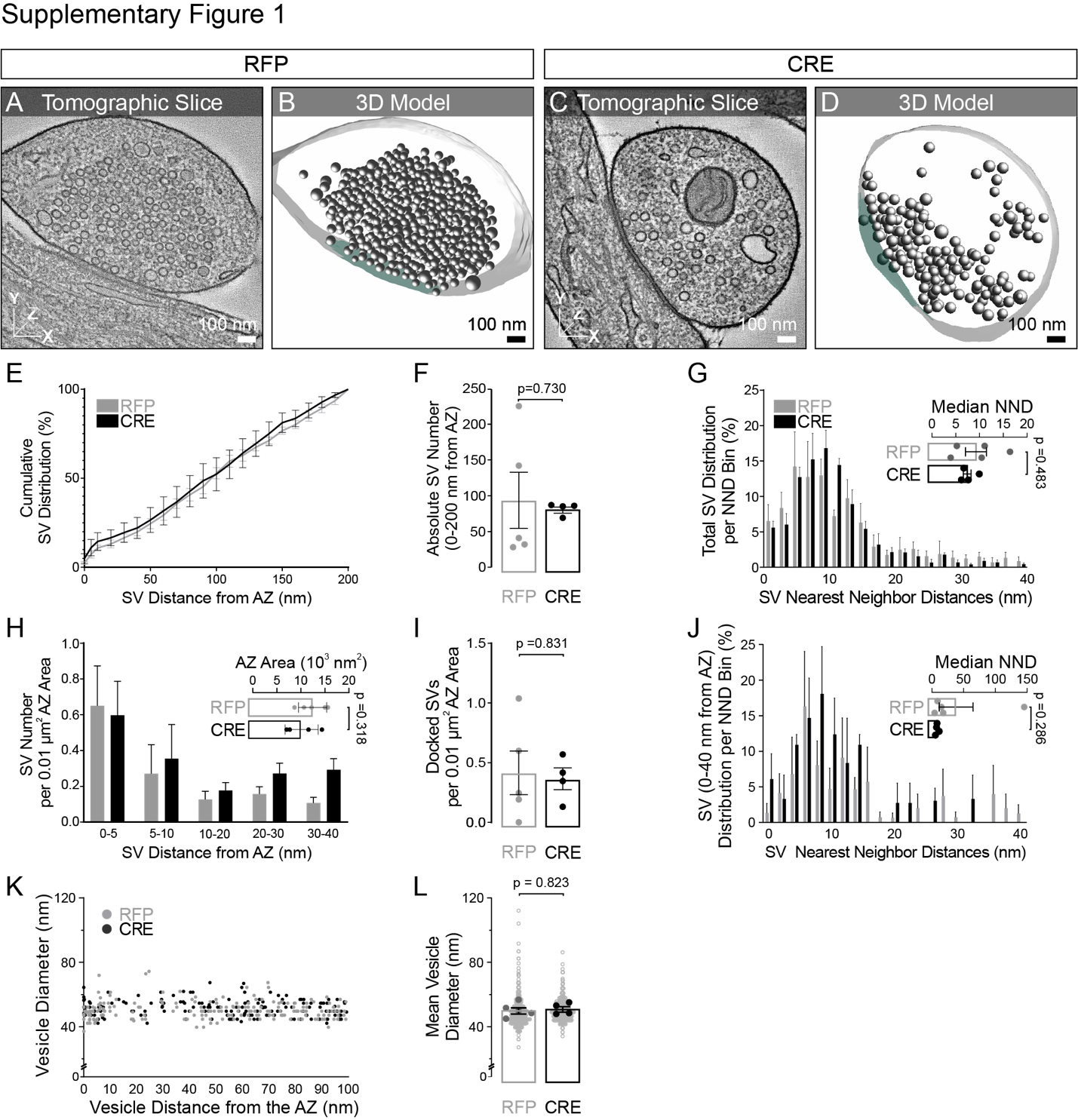


## Supplementary Figure 1: Comparative 3D ultrastructural analysis of Nedd8-deficient (CRE) and littermate control (RFP) synapses.

## A and B. Tomographic slices through representative control (A, RFP) and Nedd8-deficient (C, CRE) presynaptic boutons. B and D. Corresponding 3D models. The AZ is marked in light green. Scale bar: 100 nm.

E. Cumulative plot depicting the relative SV distribution within 0-200nm of the AZ.

F. Scatter plot indicating total SV number quantified within 0-200 nm of the AZ. p=0.730 via Mann-Whitney test.

G. Histogram showing the distribution of inter-vesicular nearest-neighbor distances (NND) quantified from all reconstructed SVs (bin size=2 nm). NND values exceeding the 0-40 nm range were not plotted. Inset graph depicts median NND values. p=0.483 with Welch’s t-test.

H. Histogram showing the spatial distribution of SVs within 0-40 nm of the AZ. Values were normalized to corresponding AZ areas. Inset graph depicts AZ areas quantified for the two conditions. p=0.318 with unpaired t-test.

I. Scatter plot indicating the number of docked SVs normalized to AZ area. p=0.831 with unpaired t-test.

J. Histogram showing the distribution of inter-vesicular nearest-neighbor distances (NND) for SVs within 0-40 nm of the AZ. Inset graph depicts median NND values quantified for the two conditions. p=0.286 with Mann-Whitney test.

K. Scatter plot relating SV diameters with respect to the spatial SV distribution within 0-100 nm of the AZ.

L. Scatter plot indicating mean SV diameter for all SVs reconstructed within the presynaptic subvolume. p=0.823 with unpaired t-test.

n=5 tomograms for RFP (control); n=4 tomograms for CRE (Nedd8 deficient).


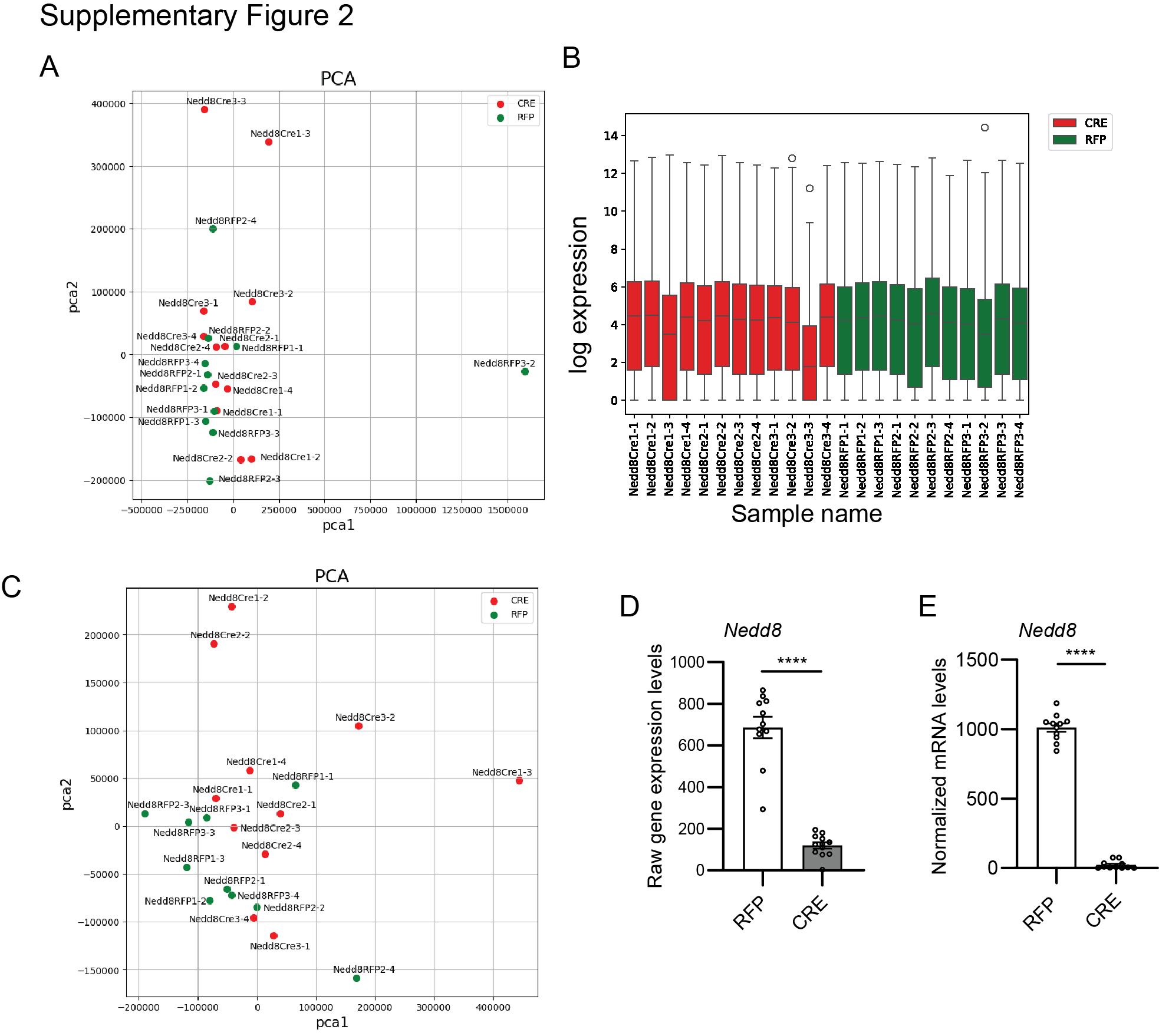


Supplementary Figure 2: Quality check for the RNA-seq. PCA (A and C) and total gene counts (B). Raw *Nedd8* gene expression (D) and normalized mRNA levels (E). Bars represent mean ±SEM. Data were compared using a Mann-Whitney test. **p<0.01, ***p<0.001 (RFP=11, CRE=11).


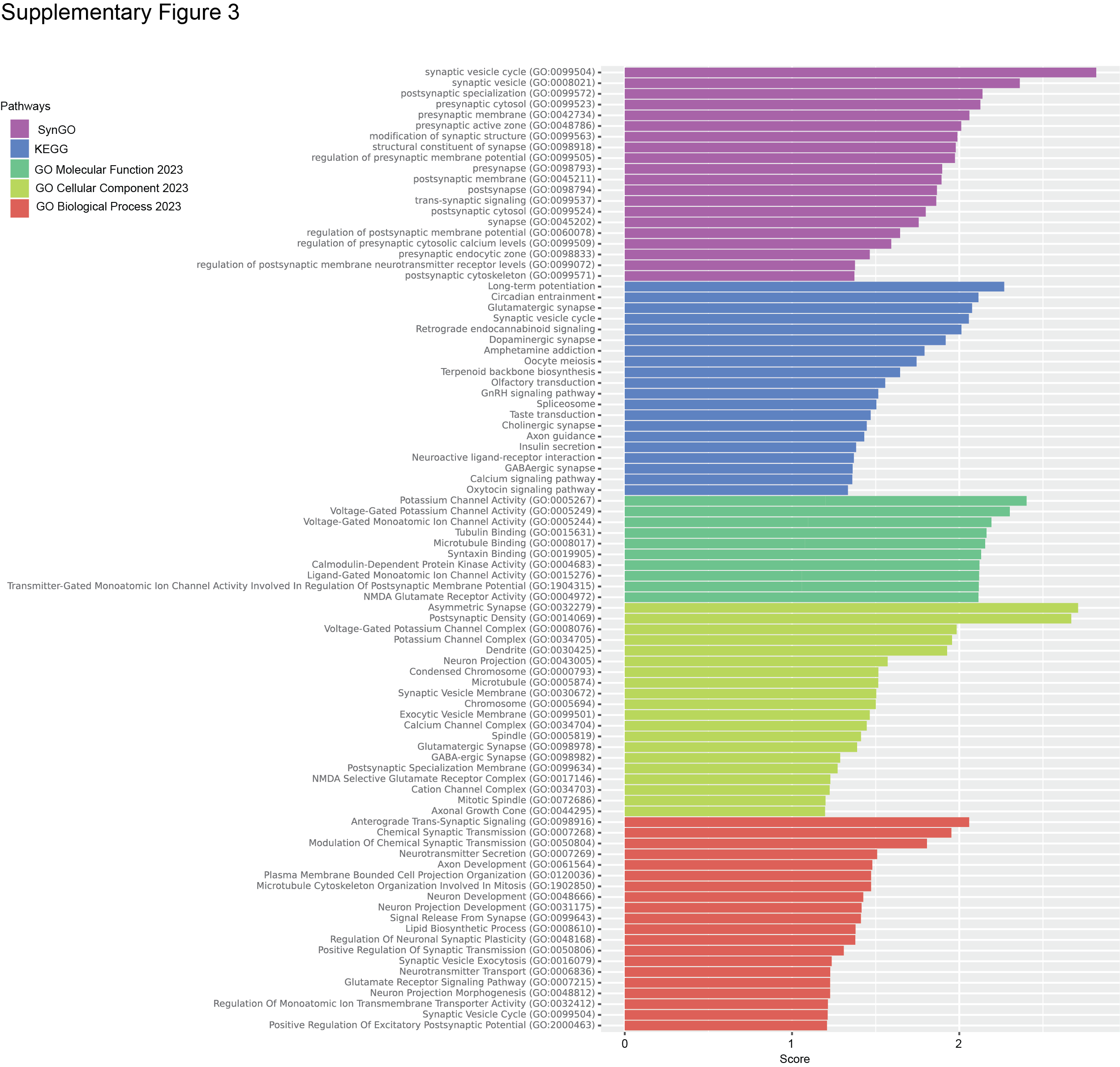


## Supplementary Figure 3: Gene ontology of down-regulated genes upon Nedd8 depletion.


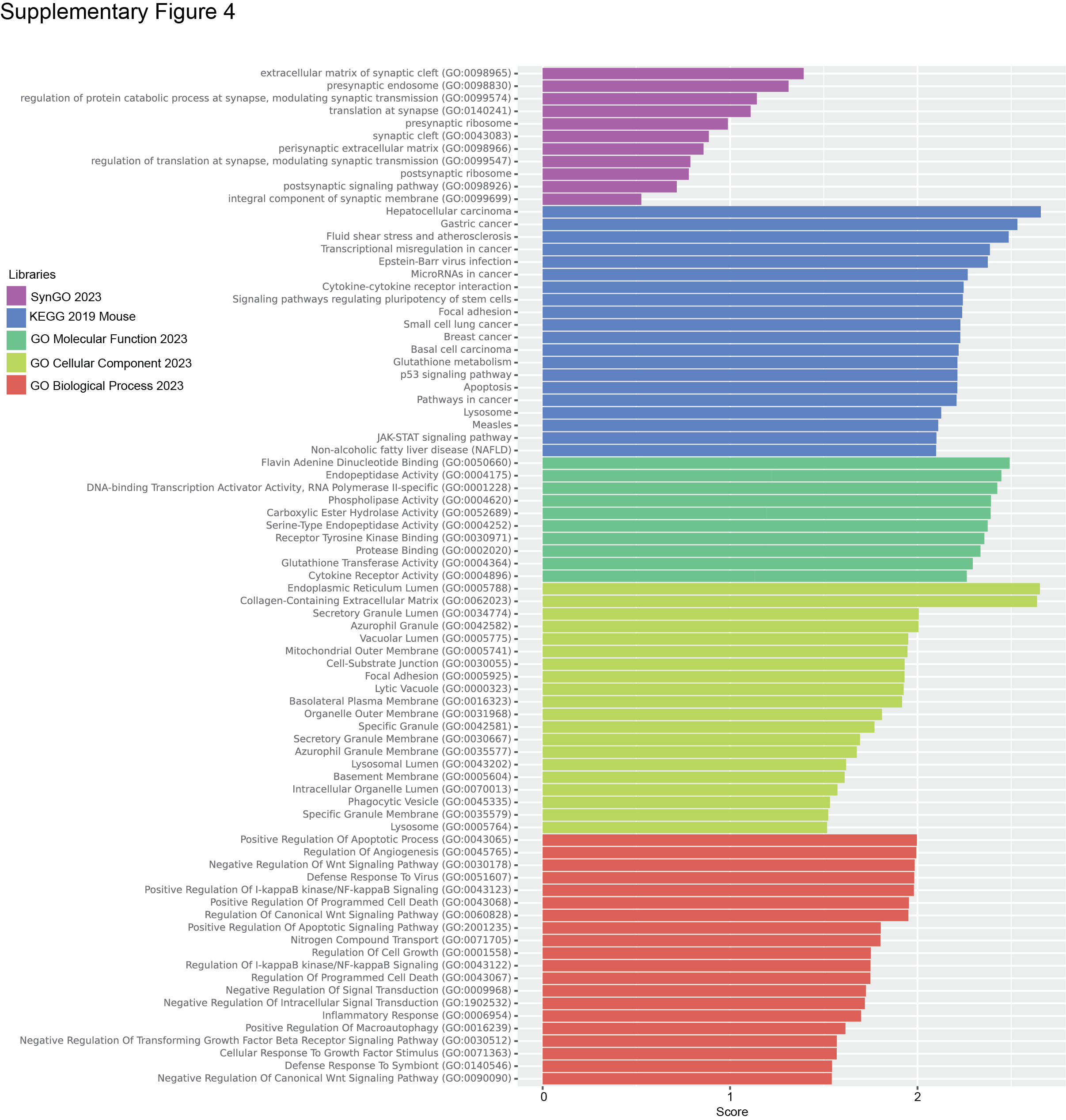


## Supplementary Figure 4: Gene ontology for up-regulated genes upon Nedd8 depletion.


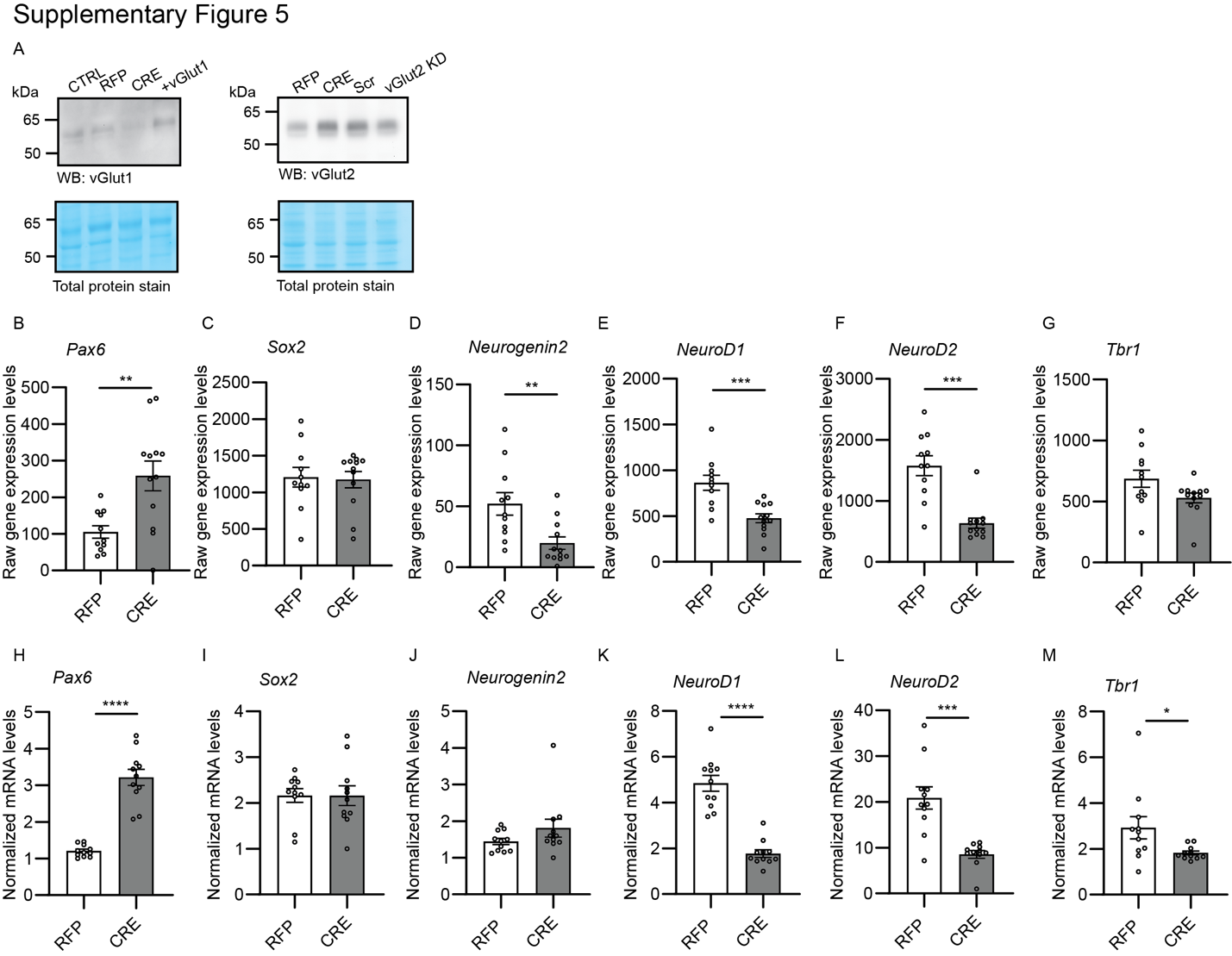


## Supplementary Figure 5: Re-expression of vGlut1 or knock-down of vGlut2 fail to rescue endophilin1 expression levels.

A. Total protein stain (bottom panels), anti-vGlut1 (left) and anti-vGlut2 (right) Western blot analysis of Nedd8cKO primary hippocampal neurons lysates infected with viruses as described above. Molecular weight is indicated on the left side (kDa).

B-G. Bar graphs showing the raw gene expression of genes as indicated on top (N=3, n_RFP_=11, n_CRE_=11).

H-M. Bar graph showing the normalized mRNA levels of each gene as indicated on top, as measured via qPCR (N=3, n_RFP_=11, n_CRE_=11).

Bars represent mean ±SEM. Data were compared using an unpaired T-test or a Mann-Whitney comparison test, where *p<0.05, **p<0.01, ***p<0.001, ****p<0.0001.


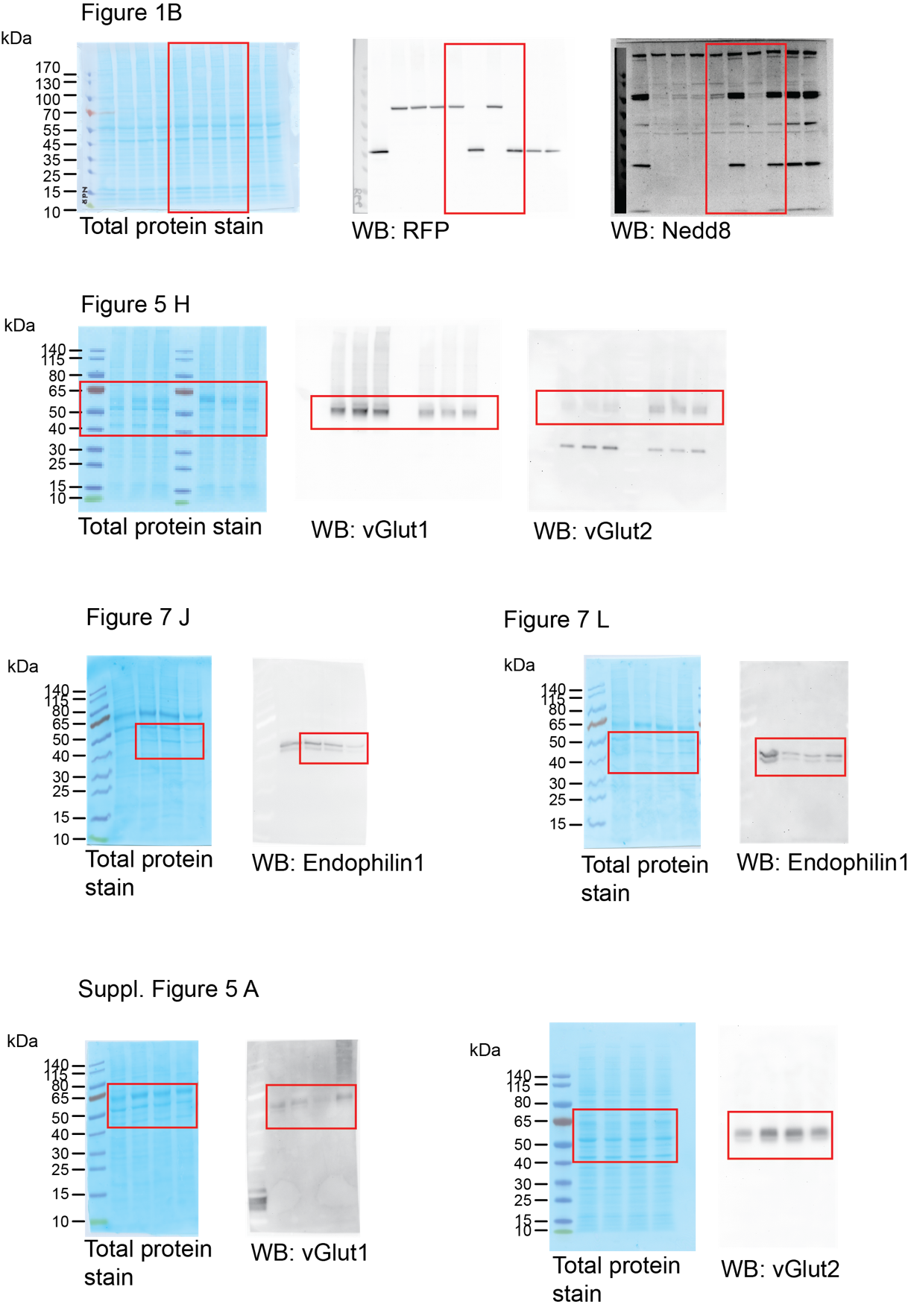
Supplementary Figure 6: original, uncropped blots for the figures as indicated on top of each panel.
