# Supplementary material for: Neddylation regulates the development and function of glutamatergic neurons
Source: Commun Biol. 2025 Sep 9;8:1338. doi: 10.1038/s42003-025-08680-x (PMC12420789; doi:10.1038/s42003-025-08680-x)
Supplement: Supplementary file 2 — Description of additional supplementary file [file 42003_2025_8680_MOESM2_ESM.docx]

## Description of Additional Supplementary File

## Supplementary Data 1: Key Resources Table: list of reagents, antibodies and oligonucleotides.

## Supplementary Data 2: List of differentially expressed genes (DE), list of differentially expressed genes after filtering (DE_filtered), list of differentially expressed genes found in the SynGO database (DE_filtered_syngo).

Supplementary Data 3: Numerical data for the confocal and STED experiments

Supplementary Data 4: Numerical data for the electrophysiological recordings

Supplementary Data 5: Numerical data for the RNA-seq, Western blot and qPCR analysis

Supplementary Data 6: Numerical data for Supplementary Figure 1

Supplementary Data 7: Numerical data for Supplementary Figure 2, 3 and 4

## 
