# Supplementary material for: Neddylation regulates the development and function of glutamatergic neurons
Source: Commun Biol. 2025 Sep 9;8:1338. doi: 10.1038/s42003-025-08680-x (PMC12420789; doi:10.1038/s42003-025-08680-x)
Supplement: Supplementary file 3 — Supplementary data 1 [file 42003_2025_8680_MOESM3_ESM.docx]

Neddylation regulates the development and function of glutamatergic neurons

Josefa Torres^1^, Zehra Vural^1^, Maksims Fiosins^2^, Valentin Schwarze^1^, Inés Hojas-García-Plaza^1^, Fritz Benseler^1^, Stefan Bonn^2^, Silvio O. Rizzoli^3^, Benjamin H. Cooper^1^, JeongSeop Rhee^1^, Nils Brose^1^, Marilyn Tirard^1^

^1^Department of Molecular Neurobiology, Max Planck Institute for Multidisciplinary Sciences, Göttingen, Germany

^2^Institute of Medical Systems Biology, Center for Biomedical AI (bAIome), Center for Molecular Neurobiology (ZMNH), University Medical Center Hamburg-Eppendorf, Hamburg, Germany

^3^Department for Neuro- and Sensory Physiology, University Medical Center Göttingen, Göttingen, Germany

Key resources table

| REAGENT or RESOURCE | SOURCE | IDENTIFIER |
| --- | --- | --- |
| Antibodies | | |
| MAP2 | Novus Biologicals | NB300 213 |
| RFP | Synaptic Systems | 390 004 |
| Synapsin-1 | Synaptic Systems | 135 302 |
| PSD95 | NeuroMab | 75028 |
| vGlut2 | Synaptic Systems | 135304 |
| Nedd8 | Cell Signaling | 2745S |
| vGlut1 | Synaptic Systems | 135 302 |
| vGlut2 | Synaptic Systems | 135 304 |
| Endophilin1 | Synaptic Systems | 159 002 |
| VAMP1/2/3 | Synaptic Systems | 104 011 |
| VAMP2 | Synaptic Systems | 104 211 |
| Synaptotagmin1 | Synaptic Systems | 105 011 |
| Syntaxin1 | Sigma | S0664 |
| Munc13-1 | Synaptic Systems | 126 103 |
| Munc13-2 | Synaptic Systems | 126 205 |
| Complexin1/2 | Synaptic Systems | 122 004 |
| SNAP25 | Synaptic Systems | 111 002 |
| Shank2 | Synaptic Systems | 162 204 |
| anti-Chicken 403 | Abcam | AB175 674 |
| anti-Guinea pig 555 | ThermoFisher | A-21435 |
| anti-Rabbit 488 | ThermoFisher | A-21206 |
| anti-Mouse 633 | ThermoFisher | A-21052 |
| anti-Guinea pig 488 | ThermoFisher | A-11073 |
| anti-Mouse-HRP | Jackson ImmunoResearch | 115-035-146 |
| anti-Guinea pig-HRP | Jackson ImmunoResearch | 106-035-003 |
| anti-Rabbit-HRP | Jackson ImmunoResearch | 111-035-144 |
| vGlut1-AZD568 | Nanotag | N1602 |
| PSD95-STAR635 | Nanotag | N3702 |
| Synaptotagmin1-ATTO488 | Nanotag | N2302 |
|  |  |  |
| Virus strains | | |
| pf(syn)-NLS-iCRE-RFP | Produced in the department | 491 |
| pf(syn)-NLS -RFP | Produced in the department | 490 |
| pAAV407-Syn-NLS-GFP-P2A—Vglut1-WPRE-hGA-pA | Produced at the Virus Core Facility, Charité Universitätsmedizin Berlin | BA-407a |
| f(u6)sgw VGlut2#1 | Virus Core Facility, Charité Universitätsmedizin Berlin | BL-113 |
| f(u6)sgw (empty) | Virus Core Facility, Charité Universitätsmedizin Berlin | HL-136 |
| Chemicals, peptides, and recombinant proteins | | |
| DMEM | Gibco | 31966021 |
| Neurobasal A | Gibco | 10888-022 |
| B27 | Gibco | 17504-044 |
| Penicillin-Streptomycin | Gibco | 15140-122 |
| Poly-L-Lysine | Sigma | P4707 |
| Lipofectamine 2000 | Thermo Scientific | 11668030 |
| Optimem | Gibco | 31985062 |
| Tetradotoxin | Tocris | 1078 |
| Glutamate | Sigma | G5889 |
| GABA | Sigma | 906818 |
| Nimodipine | Alomone Labs | N-150 |
| ω-Agatoxin | Alomone Labs | STA-500 |
| EGTA-AM | Invitrogen | E1219 |
| Triton X-100 | Roche | 40139421 |
| Bradford | Biorad | 5000006 |
| Goat serum | Gibco | 16210072 |
| Agua-Poly Mount | Polysciences Inc | 18606-5 |
| Bovine serum albumin | Biomol | 01400 |
| Mowiol | Carl Roth | 0713.1 |
| Fetal bovine serum | Gibco | 10500-064 |
| Critical commercial assays | | |
| MemCode Reversible Protein Stain Kit | Thermo Scientific | 24580 |
| Deposited data | | |
| RNA seq | Gene Expression Omnibus GEO | GSE269898 |
| Software and algorithms | | |
| PatchMaster | HEKA electronics | Version 2x90.3 |
| Axograph | Axograph Scientific | Version 1.5.4 |
| Fiji | NIH | ImageJ 2.14.0/1.54f |
| Imaris | Oxford Instruments | Version 9.9.1 |
| Mathlab | The Mathworks Inc. | Version R2022b |
| FastQC | Babraham Bioinformatics | Version 0.11.5 |
| GraphPad Prism | GraphPad | Version 10.3.0 |

All oligonucleotides were locally produced at the DNA core facility of the department of the Max Planck Institute for Multidisciplinary Sciences.

| OLIGONUCLEOTIDES | SEQUENCE |
| --- | --- |
| Nedd8-protospacer-sgRNA1 (upstream Nedd8_Exon2) | 5’- AACCTCAGTCCACAAGCATG-3’ (PAM = GGG) |
| Nedd8-protospacer-sgRNA2 (downstream Exon3) | 5’- CTGCGAAAGAGCGGCAAGCA-3’ (PAM = AGG) |
| s_Up_HDR1_Nedd8-cKO | 5 -CTAGGCCAAGTGTGG-TGTTTGTGGT-3' |
| as_Down_HDR1_Nedd8-cKO | 5'-AATCCCAGCAGACAAATCCATGTGA-3' |
| Sense2_Nedd8_UpExon2 | 5'-CGTATTTAATTTGGAGCTTACCGTT-3' |
| Asense3_Nedd8_UpExon2 | 5'-GCAAAGATGGCGTAAGTCCCACA-3' |
| Asense_Nedd8_DownExon3 | 5'-GGCACCTGTACTCAAGCGTA-3' |
| Forward_GAPDH | 5'-ATCTCCACTTTGCCACTGC -3' |
| Rerverse_GAPDH | 5'-AGGTCGGTGTGAACGGATTTG -3' |
| Forward_UbcC | 5'-CCACACAAAGCCCCTCAAT -3' |
| Rerverse_UbcC | 5'-CAAAGATCTGCATCGTCTCTCTC -3' |
| Nedd8_Sense_Exon1 | 5’-AGCACTCTAGCCGCCTGCAA-3’ |
| Nedd8_Asense_Exon2_3 | 5’-TCCTTGATTCGCTCCACCT-3’ |
| Sense_Nedd8_Exon2_Exon3 | 5’-GAACCCACAGACAAGGTGGA-3’ |
| Asense_Nedd8_Exon4 | 5’-GGACGGAACCACCTAGAATCT-3’ |
| Sense_NeuroD1 | 5'-CCGAGGCTCCAGGGTTATGA-3', |
| Asense_NeruoD1 | 5’-CATCAGCCCGCTCTCGCTGT-3’ |
| Sense_Neurod1_upstream_STOP | 5’-GATAGCCATTCGCATCATGAGC-3’ |
| Asense_Neurod1_downstream | 5`-TGCCTTCTGTAAACAGGACAGTCA-3’ |
| Sense_NeuroD2_1 | 5'-TCAAGGAGGAAGGAGAGCTG-3' |
| Asense_NeruoD2_1 | 5'-GGTCATCTTGCGTTTCTTCG-3' |
| Sense_NeuroD2_2 | 5'-TCTCTCGGAGATCTTGCGCT-3' |
| Asense_NeruoD2_2 | 5’-TGCACAGAGTCTGCACGTAGG-3' |
| Sense_NeuroD2_3 | 5'-CATGTACGAGGAGCTCAACGC-3' |
| Asense_NeruoD2_3 | 5'-CCCACAGGTAAGAGAGCACG-3' |
| Sense_Neurog2_Exon1_Exon2 | 5’-ATCTGGAGCCGCGTAGGATG-3’ |
| Asense_Exon2 | 5’-GAAGCCGAGCCCAGCAGCAT-3’ |
| Sense_Neurog2_2 | 5'-TCAAGAAGACCCGCAGGCT-3' |
| Asense_Exon2_2 | 5'-GTTTAGGTTGTGCATGCGGTT-3' |
| Sense_Neurog2_3 | 5'-CGGGTCAGACGTGGACTACT-3' |
| Asense_Exon2_3 | 5'-GGTGAGGCGCATAACGAT-3' |
| Sense_Pax6 | 5'-ACTTCAGTACCAGGGCAACC-3' |
| Asense_Pax6 | 5'-GAACTGATGGAGTTGGTGTTCTC-3' |
| Asense_Pax6_Exon9_Exon10_transv1 | 5'-CTGAAGCGGAAGCTGCAAAG-3' |
| Sense_Pax6_Exon9_Exon10_transv1 | 5’-ATGGGTCCTCTCAAACTCTTTC-3’ |
| Sense_Sh3gl2_Exon3 | 5’-GGGCTGTGATGGAGATAATGA-3’ |
| Asense_Sh3gl2_Exon4 | 5’-CCTTCTCTTGGCCGCGGAT-3’ |
| Sense_Sh3gl2_2 | 5'-GCACACCCAAACCTCCAG-3' |
| Asense_Sh3gl2_2 | 5'-TTCAGGTTCAAAGTCATACAGAGC-3' |
| Sense_Slc17a6_Vglut2_Exon3_transv1 | 5’-ATTCCAGGAGGATATATCGCAT-3’ |
| Asense_Slc17a6_Vglut2_Exon4_transv1 | 5’-ACATCCATAATGCACTCTGGCT-3’ |
| Sense_Slc17a6_Vglut2_2 | 5'-TGGCTACAACCTCCTTTTGTG-3' |
| Asense_Slc17a6_Vglut2_2 | 5'-GGATACCAGCTAAGGGCATTG-3' |
| Sense_Slc17a7_vGlut1_1 | 5’-CACGTGGTGGTGCAGAAA-3' |
| Asense_Slc17a7_vGlut1_1 | 5'-CCTCCAGGAATCTGAGTGACA-3' |
| Sense_Slc17a7_vGlut1_2 | 5'-ACAGCCTTTTGCGGTTCCTA-3' |
| Asense_Slc17a7_vGlut1_2 | 5'-GCAGTGCCGGTGACTCATAG-3' |
| Sense_Slc17a7_vGlut1_3 | 5'-GTGCAATGACCAAGCACAAG-3' |
| Asense_Slc17a7_vGlut1_3 | 5’-TAGTGCACCAGGGAGGCTAT-3' |
| Sense_Sox2_CDS | 5’-CCGTTCATCGACGAGGCCAA-3’ |
| Asense_Sox2_CDS | 5'-CGGGAAGCGTGTACTTATCCTT-3' |
| Sense_Sox2_3'-UTR | 5’-GGCTGGACTGCGAACTGGA-3’ |
| Asense_Sox2_3'-UT | 5’-TGTCGTTTCGCTGCGGAGATT-3’ |
| Sense_Tbr1_1 | 5'-CAAGGGAGCATCAAACAACA-3' |
| Asense_Tbr1_1 | 5’-GTCCTCTGTGCCATCCTCAT-3' |
| Sense_Tbr1_2 | 5’-CCGAGTCCAGACGTTCACTT-3' |
| Asense_Tbr1_2 | 5'-GCCCGTGTAGATCGTGTCAT-3' |
| Sense GAPDH | 5'-ATCTCCACTTTGCCACTGC -3' |
| Antisense GAPDH | 5'-AGGTCGGTGTGAACGGATTTG -3' |
| Sense Ubc | 5'-CCACACAAAGCCCCTCAAT -3' |
| Antisense Ubc | 5'-CAAAGATCTGCATCGTCTCTCTC -3' |
| Nedd8-Exon2-Exon3-floxed_HDR fragment  CDS Exon2, Exon3 & Stop-Exon4 = **BLACK BOLD UPPER CASE**  loxP1 & loxP2 = **BLUE BOLD UPPER CASE**  STOP Codon TGA = **RED BOLD UPPER CASE**  3’-UTR = underlined | 5'-taagagcacataccgaagccgttgggcacactcctaaaatcacagcagttagagaaagctgagacaggaaaattacaagggtgagttccaggccagtctaggctgcctagtcaaagcaatacaaaagatgatgggtggattagaggttgtgtgaatcacagatttacagtttgtacacaaatatatttggttacctagttttagcctgtggaaatcaagcaatttgagttggagtttaggatttagatagaattctgggacactaggaaagaatataatgttcaactctagcatttcaaaaaaaatctagttggtggtgattacttgtgcccttgaaaactctttgcattatataaatgtaatttgtgccctgggataaagtccttactttaggattgcttaacctagttcagttgtcattgcatctcatccaaagaattcctgatattcttgctgacttggggtgggggtggggaacggcttgtggcattaaggggatcaaatgggttcgcagaatgctgtcttacactgcctgatgacatgggctgtggcacagccttctgattcttcagggtagtattgctgagaatatatacataacaactctcagcagagagaaccccagagaccaagataagatgttacacttagaacatgtcttgccaggcaatggtagtgcatacctttaatcccagcacttggaaggcaaagatcagggccacacaaaataaaccctgtcaccaaaaaaaaaaaattcttctatttttgtgaaaagacactatgataaaagaacgtatttaatttggagcttaccgtttcagaggggaagtccaatactatcatggcagagaggatggcagcagacaggtaggcatggttctggagcagaagctaagagcttaaacctcagtccaca**ATAACTTCGTATAATGTATGCTATACGAAGTTAT**agcatgaggcagagaaagagaacatggcttgggcttttgaagcctccaacaagaccacacctctaatacttcccgttccaccaactgtgtgccatgtattcaaacatgagcctattctcattcagtccaccacagaaagcaccttggtatcgggctgtgggacttacgccatctttgcctgttttag**ACGCTGACTGGGAAGGAGATTGAGATAGACATCGAACCCACAGACAAG**gtgagtcaggtgctcagcgtcctttctctgtatgtgtgcttgcatgtgtgtgcttgcatgtgcttatgaccttggtcctttccttctcaggttgtctgtctatacagcctttgttcttagcattggtttgtttgtttgtttgtttattttagttttccgagacagagtttctctctgtgtacccctggctgtcctggaactcagactggccttgaattcatagagagcagcctgtctctaccttccaagtgctgggactaaaggctagtatttgtctaattctaagcagcctctgaccacttgtttctccaaag**GTGGAGCGAATCAAGGAGCGTGTGGAAGAAAAAGAAGGGATTCCCCCCCAGCAGCAGCGGCTCATCTACAGTGGCAAGCAAAT**gtaagcttgggtgggaagtgaggtatagctgccttgtgtgaggatgagtgcaggggaaggtgacgaggctacaggttcatcgggcctgtgctgcttcagtgtgtccctgcttg**ATAACTTCGTATAATGTATGCTATACGAAGTTAT**ccgctctttcgcagtctgatgaagcatatagaatcttctcagaatataagagaaagcatagaatattccagacttagagtaaaatagattggattgtaaactaatattgagctagttaacaaactactaaaacaaatttgtagaatagtaatatatgtgcttttatatgcctttaaattgattatgttcaggtatgtatttataggtatatacgcttgagtacaggtgccctcagatttcagagctgtcagatcctcctggaaccttatttaaatgagattgtgagcctcctgaagtgggtactgggacctgaactcaggtcctctgcaagagaagcaatttgagcttttaacttctgagccatctttccagccctccttattaacatcttattagaaggtctaatgattagtctgataattgcttttttcttgtttttacattttcaagatacttgcaaatagtcataatgtgatatcatctaatttctattgtgtaagctatcatattgttaacactgtgggtctctgtattcaaaattaaatactaattacccttagaggttagtaaaagaatatttttctcatacagtttcataggcctctcagagatccaccaaactcaggttacaaatgcccattttagatggatttctaatgctttttcttctctgtag**GAATGATGAGAAGACAGCAGCTGATTACAAGATTCTAGGTGGTTCCGTCCTCCACCTGGTGTTGGCTCTTAGAGGAGGAGGTGGTCTTGGGCAGTGA**agaaacttggttccgtttacctccttgccctgccaatcataatgtggcatcacatatcctctcactctctgggacaccagagccactgccccctctcttggatgcccaatcttgtgtgtctactggtgggagaatgtgaggaccccagggtgcagtgttcctggcccagatggcccctgctggctattgggttttagtttgcagtcatgtgtgcttccctgtcttatggctgtatccttggttatcaataaaatatttcctggccatctggactctttcttcttgacacaaataactgaaatccaagtggctataaccagtagaagtagaacggtggaaggaaaggtgctgctcttaggttagttttgagaaggtgtgggtgggatagg -3' |
