# Supplementary material for: Neddylation regulates the development and function of glutamatergic neurons
Source: Commun Biol. 2025 Sep 9;8:1338. doi: 10.1038/s42003-025-08680-x (PMC12420789; doi:10.1038/s42003-025-08680-x)
Supplement: Supplementary file 10 — Reporting Summary [file 42003_2025_8680_MOESM10_ESM.pdf]

nature portfolio

Corresponding author(s): Marilyn Tirard

Last updated by author(s): 2025/05/28

Reporting Summary

Nature Portfolio wishes to improve the reproducibility of the work that we publish. This form provides structure for consistency and transparency in reporting. For further information on Nature Portfolio policies, see our Editorial Policies and the Editorial Policy Checklist.

Statistics

For all statistical analyses, confirm that the following items are present in the figure legend, table legend, main text, or Methods section.

n/a	Confirmed	
☐	☒	The exact sample size (n) for each experimental group/condition, given as a discrete number and unit of measurement
☐	☒	A statement on whether measurements were taken from distinct samples or whether the same sample was measured repeatedly
☐	☒	The statistical test(s) used AND whether they are one- or two-sided<br>*Only common tests should be described solely by name; describe more complex techniques in the Methods section.*
☐	☒	A description of all covariates tested
☒	☐	A description of any assumptions or corrections, such as tests of normality and adjustment for multiple comparisons
☒	☐	A full description of the statistical parameters including central tendency (e.g. means) or other basic estimates (e.g. regression coefficient) AND variation (e.g. standard deviation) or associated estimates of uncertainty (e.g. confidence intervals)
☒	☐	For null hypothesis testing, the test statistic (e.g. F, t, r) with confidence intervals, effect sizes, degrees of freedom and P value noted<br>*Give P values as exact values whenever suitable.*
☒	☐	For Bayesian analysis, information on the choice of priors and Markov chain Monte Carlo settings
☒	☐	For hierarchical and complex designs, identification of the appropriate level for tests and full reporting of outcomes
☒	☐	Estimates of effect sizes (e.g. Cohen's d, Pearson's r), indicating how they were calculated

Our web collection on statistics for biologists contains articles on many of the points above.

Software and code

Policy information about availability of computer code

Data collection	n/a
Data analysis	n/a

For manuscripts utilizing custom algorithms or software that are central to the research but not yet described in published literature, software must be made available to editors and reviewers. We strongly encourage code deposition in a community repository (e.g. GitHub). See the Nature Portfolio guidelines for submitting code & software for further information.

Data

Policy information about availability of data

All manuscripts must include a data availability statement. This statement should provide the following information, where applicable:

- Accession codes, unique identifiers, or web links for publicly available datasets
- A description of any restrictions on data availability
- For clinical datasets or third party data, please ensure that the statement adheres to our policy

RNA-seq data are accessible under the GSE269898 and are publicly available as of the date of publication.
Data described in this study, such as Western blot or microscopy data, will be shared upon request by the lead contact. Any additional information required to reanalyse the data reported in this work is available from the lead contact upon request.

This paper does not report original code. Jupiter Notebook for graph generation can be shared upon request.

Research involving human participants, their data, or biological material

Policy information about studies with human participants or human data. See also policy information about sex, gender (identity/presentation), and sexual orientation and race, ethnicity and racism.

Reporting on sex and gender	n/a
Reporting on race, ethnicity, or other socially relevant groupings	n/a
Population characteristics	n/a
Recruitment	n/a
Ethics oversight	n/a

Note that full information on the approval of the study protocol must also be provided in the manuscript.

Field-specific reporting

Please select the one below that is the best fit for your research. If you are not sure, read the appropriate sections before making your selection.

☒ Life sciences ☐ Behavioural & social sciences ☐ Ecological, evolutionary & environmental sciences

For a reference copy of the document with all sections, see nature.com/documents/nr-reporting-summary-flat.pdf

Life sciences study design

All studies must disclose on these points even when the disclosure is negative.

Sample size	sample size calculation was not performed
Data exclusions	no data points were excluded
Replication	we have multiple biological replicates that ensure for reproducibility
Randomization	n/a
Blinding	technical limitations and the nature of the analysis prevented bliding

Reporting for specific materials, systems and methods

We require information from authors about some types of materials, experimental systems and methods used in many studies. Here, indicate whether each material, system or method listed is relevant to your study. If you are not sure if a list item applies to your research, read the appropriate section before selecting a response.

Materials & experimental systems

n/a	Involved in the study	
☐	☒	Antibodies
☐	☒	Eukaryotic cell lines
☒	☐	Palaeontology and archaeology
☐	☒	Animals and other organisms
☒	☐	Clinical data
☒	☐	Dual use research of concern
☒	☐	Plants

Methods

n/a	Involved in the study	
☒	☐	ChIP-seq
☒	☐	Flow cytometry
☒	☐	MRI-based neuroimaging

Antibodies

Antibodies used	MAP2 Novus Biologicals NB300 213

Antibodies used

RFP Synaptic Systems 390 004
Synapsin-1 Synaptic Systems 135 302
PSD95 NeuroMab 75028
vGlut2 Synaptic Systems 135304
Nedd8 Cell Signaling 2745S
vGlut1 Synaptic Systems 135 302
vGlut2 Synaptic Systems 135 304
Endophilin1 Synaptic Systems 159 002
VAMP1/2/3 Synaptic Systems 104 011
VAMP2 Synaptic Systems 104 211
Synaptotagmin1 Synaptic Systems 105 011
Syntaxin1 Sigma S0664
Munc13-1 Synaptic Systems 126 103
Munc13-2 Synaptic Systems 126 205
Complexin1/2 Synaptic Systems 122 004
SNAP25 Synaptic Systems 111 002
Shank2 Synaptic Systems 162 204
anti-Chicken 403 Abcam AB175 674
anti-Guinea pig 555 ThermoFisher A-21435
anti-Rabbit 488 ThermoFisher A-21206
anti-Mouse 633 ThermoFisher A-21052
anti-Guinea pig 488 ThermoFisher A-11073
anti-Mouse-HRP Jackson ImmunoResearch 115-035-146
anti-Guinea pig-HRP Jackson ImmunoResearch 106-035-003
anti-Rabbit-HRP Jackson ImmunoResearch 111-035-144
vGlut1-AZD568 Nanotag N1602
PSD95-STAR635 Nanotag N3702
Synaptotagmin1-ATTO488 Nanotag N2302

Validation

done by western blot and immunostaining analysis

Eukaryotic cell lines

Policy information about cell lines and Sex and Gender in Research

Cell line source(s)

HEK293FT cell, Thermofischer

Authentication

not authenticated

Mycoplasma contamination

not tested in the current study

Commonly misidentified lines (See ICLAC register)

n/a

Animals and other research organisms

Policy information about studies involving animals; ARRIVE guidelines recommended for reporting animal research, and Sex and Gender in Research

Laboratory animals

Nedd8cKO homozygous in C57BL/6N

Wild animals

n/a

Reporting on sex

sex of the P0 animals was not taken into account in the current study

Field-collected samples

n/a

Ethics oversight

Mouse maintenance and breeding were performed with permission of the Niedersächsisches Landesamt für Verbraucherschutz und Lebensmittelsicherheit (LAVES).

Note that full information on the approval of the study protocol must also be provided in the manuscript.

Plants

Seed stocks	n/a
Novel plant genotypes	n/a
Authentication	n/a
